# Design of Distributed Engine Control Systems with Uncertain Delay

**DOI:** 10.1371/journal.pone.0163545

**Published:** 2016-09-26

**Authors:** Xiaofeng Liu, Yanxi Li, Xu Sun

**Affiliations:** 1 School of Transportation Science and Engineering, Beihang University, Beijing, PR China; 2 Collaborative Innovation Center for Advanced Aero-Engine, Beijing, PR China; 3 Aircraft/Engine Integrated System Safety Beijing Key Laboratory, Beijing, PR China; 4 School of Energy and Power Engineering, Beihang University, Beijing, PR China; West Virginia University, UNITED STATES

## Abstract

Future gas turbine engine control systems will be based on distributed architecture, in which, the sensors and actuators will be connected to the controllers via a communication network. The performance of the distributed engine control (DEC) is dependent on the network performance. This study introduces a distributed control system architecture based on a networked cascade control system (NCCS). Typical turboshaft engine-distributed controllers are designed based on the NCCS framework with a *H*_∞_ output feedback under network-induced time delays and uncertain disturbances. The sufficient conditions for robust stability are derived via the Lyapunov stability theory and linear matrix inequality approach. Both numerical and hardware-in-loop simulations illustrate the effectiveness of the presented method.

## Introduction

A distributed control system (DCS) is a control system, wherein the control elements are distributed throughout the system, unlike centralized ones, where only a single controller at a central location is used. In a DCS, a hierarchy of controllers is connected by communication networks for information transmission. The advantages of the DCS architecture, such as system weight reduction, higher reliability, modularity, and less maintenance costs, merit increasing attention from industrial companies and engineers.

Conventional gas turbine engine control systems are designed as a centralized architecture called the full authority digital engine control (FADEC)to protect the control elements from extreme environment [1]. With the increasing development of sophisticated electronics in gas turbine engine control systems, increased performance, more convenient operation, and reduction of design and maintenance costs require a more effective architecture for the control systems; hence, the development of the DEC architecture [2].

The sensors and controllers are connected by communication networks and between the controllers and the actuators because of the distributed architecture [3]. The DEC architecture can be viewed as an NCCS. For example, the GE T700 turboshaft engine is a two-spool engine consisting of a gas generator and a free power turbine [4] [5]. The power turbine is connected to the rotor system by a shaft and a gearbox. The power turbine can be conventionally considered as a part of the rotor system [6]. The rotor system input is the gas generator’s output, which is the shaft torque. Therefore, the whole turboshaft engine system, combined with the control systems, can be reviewed as a cascade control system (CCS) [7].

Fundamental factors affect the DEC system that uses the communication network to close the control loop. They include network-induced time delay, packet dropouts, and bandwidth constraints [8] [9]. Hence, the control system should be robust to these factors to guarantee the desired performance and ensure stability. The network-induced time delay in the NCCSs occurs when the sensors, controllers, and actuators transfer information/data through the networks, which can degrade the performance of the control systems and even destabilize the system [10]. The network-induced time delay is unavoidable in the NCCSs. Hence, existing literature, such as [11] [12] [13] [14] and the references therein, discuss the time delay. Moreover, many useful approaches were proposed [15] [16] [17] [18] and applied to the industrial systems (see [19] [20] [21] [22] [23] and the references therein).

However, only a few studies discussed the DEC robust control in gas turbine engine control systems. Accordingly, Belapurkar et al. [24] analyzed the stability of a set-point controller for partial DEC systems with time delays by using the linear quadratic regulator (LQR) method. Yedavalli et al. [9] discussed the DEC system stability under communication packet dropouts. Merrill et al. [2] provided a DEC design approach based on quadratic invariance optimal control theory to the control performance of various types of decentralized network configurations.

The present study is concerned with the problem of the *H*_∞_ controller design for the gas turbine engine-distributed control by using an output feedback control in the form of NCCSs with uncertain delays. The rest of the paper is organized as follows: the architecture of the distributed engine control system is thoroughly described in the next section. An NCCS model of a GE T700 turboshaft engine is established and the *H*_∞_ output feedback controllers are designed based on Lyapunov stability theory and LMI approach in the following section. Simulation examples are presented in the simulation results section to illustrate the effectiveness of the approach. The conclusion is found in the last section.

## DEC System Architecture of the GE T700 Turboshaft Engine

**Fig 1:**
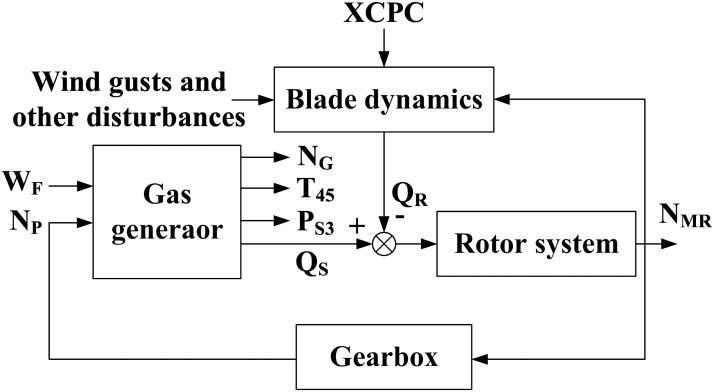
Block diagram of the open-loop gas generator/rotor system.

This study utilized a GE T700 turboshaft engine. Fig 1 shows the simplified diagram. Table 1 presents the abbreviations of the engine parameters. The inputs to the gas generator were the power turbine speed set value, *N*_*P*_, and the fuel flow rate, *W*_*F*_. The outputs were the gas generator speed, *N*_*G*_, engine torque transmitted by the power turbine shaft, *Q*_*S*_, compressor static discharge pressure, *P*_*S*3_, and power turbine inlet temperature, *T*_45_.

**Table 1:** Symbols of the GE T700 turboshaft engine.

Symbols and meaning	
*PLA*	Power lever angle (throttle)
*N*_*G*_	Gas generator speed
*N*_*P*_	Power turbine speed
*N*_*MR*_	Main rotor blade velocity
*Q*_*MR*_	Rotor torque state
*Q*_*S*_	Engine shaft torque
*XCPC*	Collective pitch
*P*_1_	Inlet pressure
*P*_*S*3_	Static pressure at Station 3
*T*_1_	Inlet temperature
*T*_45_	Inter-turbine gas temperature
*W*_*F*_	Fuel flow
*J*_*G*_	Power turbine inertia
*J*_*T*_	Lumped power turbine/dynamometer inertia
*J*_*MR*_	Main rotor blade inertia
*KMR*	Stiffness of the centrifugal restoring springs
*DMR*	Lag hinge damping
*DAM*	Aero damping
*r*	Reference input
*x*	Model state vector
*y*	Model output vector
*u*	Model input vector

**Fig 2:**
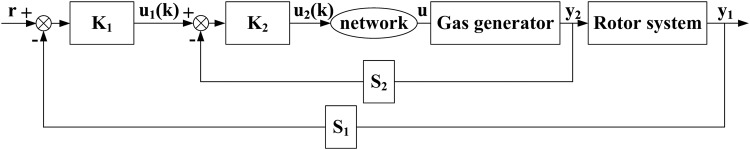
Block diagram of the NCCS model.

Control laws essentially work to maintain the power turbine speed, *N*_*P*_, constant at the set point by modulating the fuel flow, *W*_*F*_. The control accomplishes this by scheduling a nominal *N*_*G*_ speed as a function of the XCPC, *T*_1_ and *P*_1_. The control trims this *N*_*G*_ demand to isochronously adjust *N*_*P*_ to the *N*_*P*_ set input. The power lever angle (PLA) position limits the maximum permissible *N*_*G*_, while the control further limits the maximum *T*_45_. The control limits the *N*_*G*_ acceleration/deceleration rate as a function of an *N*_*G*_ scheduled *W*_*F*_/*P*_*S*3_ limit. The DEC discussed herein has one network, which is inserted in the gas generator controller and the gas generator. Fig 2 shows the architecture. The abovementioned description illustrates that the GE T700 control structure is a cascade control structure, wherein the desired primary process output can only be controlled by controlling the secondary control process output.

The following assumptions are partially taken from [25] [26]:

- The controllers are event driven. The primary controller computes the values and sends them to the secondary controller after obtaining the latest samples of the primary plant outputs. The secondary controller then computes the control command and sends it to the actuator as soon as it receives the latest samples of the secondary plant and the control output of the primary plant controller through a common network.
- The actuator is time driven. In other words, the actuator actuates the plants once it receives the control command. The actuator will then use the previous value by zero-order-hold to precede the secondary process in case of packet loss.
- The sensors are time driven, that is, they periodically sample the outputs and send them to the corresponding controllers.
- The data packet transmitted from the controller to the plant may be delayed. The delay is assumed to be a fixed one and less than a sampling period *h* (i.e., *τ*_*k*_ ∈ [0, *h*]).
- The data packet is assumed to be transmitted between the primary and secondary controllers in a single packet without any loss. However, the data packet transmitted between the secondary controller and the actuator may be delayed or may meet a possible failure in a random manner.

## Robust *H*_∞_ Output Control for GE T700

### Model description

**Fig 3:**
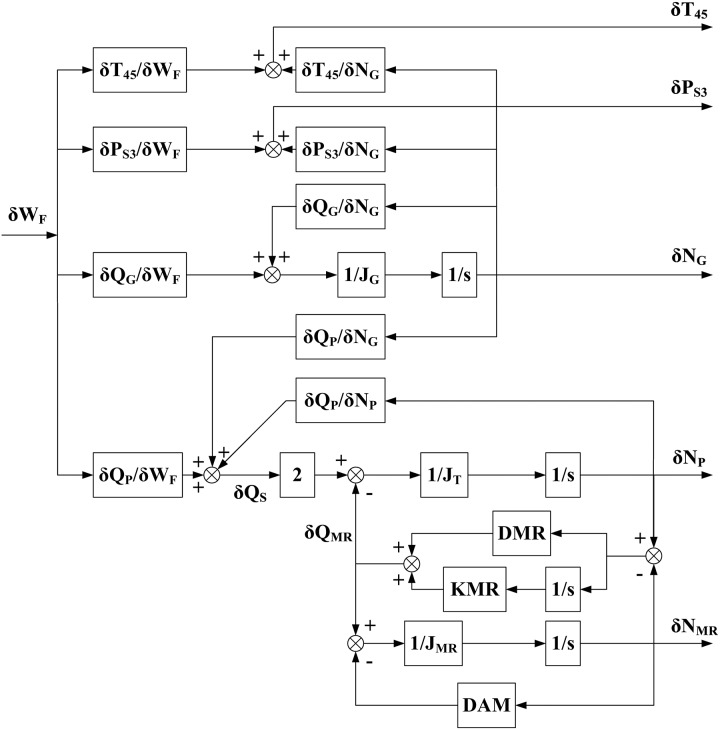
Block diagram of the simplified linearized gas generator and rotor system.

The controller design process begins with a linearized, state-space model of the system. Fig 3 shows the simplified model in this case.

**Primary plant**: The state-space representation of the rotor system is provided by the following equation:

$$\begin{array}{c} \begin{array}{cc} & {\dot{x}}_{1}\left(t\right)=A_{1}x_{1}\left(t\right)+B_{1}y_{2}\left(t\right)\\ & y_{1}\left(t\right)=C_{1}x_{1}\left(t\right) \end{array} \end{array}$$ (1)

where, *x*_1_ = [*N_P_ N_MR_ Q_MR_*]^*T*^, and *y*_1_ = *N*_*P*_ are the state vector and the output of the rotor system, respectively. *y*_2_ = *Q*_*S*_ is the gas generator output. The matrices $A_{1}$, $B_{1}$, and *C*_1_ are provided as follows:

$$A_{1}=[\begin{array}{ccc} 0 & 0 & -\frac{1}{J_{T}}\\ 0 & -\frac{DAM}{J_{MR}} & \frac{1}{J_{MR}}\\ KMR & \frac{DMR\cdot DAM}{J_{MR}}-KMR & -\frac{DMR}{J_{T}}-\frac{DMR}{J_{MR}} \end{array}],\;B_{1}=[\begin{array}{c} \frac{2}{J_{T}}\\ 0\\ \frac{2\cdot DMR}{J_{T}} \end{array}],\;C_{1}=[\begin{array}{ccc} 1 & 0 & 0 \end{array}]\text{.}$$

**Secondary plant**: The continuous-time linear model of the gas generator is shown as follows:

$$\begin{array}{c} \begin{array}{cc} & {\dot{x}}_{2}\left(t\right)=A_{2}x_{2}\left(t\right)+B_{2}u\left(t\right)+B_{3}w\left(t\right)\\ & y_{2}\left(t\right)=C_{2}x_{2}\left(t\right) \end{array} \end{array}$$ (2)

where, *x*_2_ = [*N_G_* *Q_S_* *T*_45_ *P*_*S*3_ *N_P_*]^*T*^, *y*_2_ = *Q*_*S*_ are the state and output vectors; *u*(*t*) = *W*_*F*_ is the control input; and *w*(*t*) is the exogenous disturbance signal belonging to *l*_2_[0, ∞). The matrices $A_{2}$, $B_{2}$, and *C*_2_, are presented as follows:

$$A_{2}=[\begin{array}{ccccc} \frac{1}{J_{G}}\cdot \frac{\delta Q_{G}}{\delta N_{G}} & 0 & 0 & 0 & 0\\ \frac{2\cdot DMR}{J_{T}}\cdot \frac{\delta Q_{P}}{\delta N_{G}} & 0 & 0 & 0 & \frac{2\cdot DMR}{J_{T}}\cdot \frac{\delta Q_{P}}{\delta N_{P}}\\ \frac{\delta T_{45}}{\delta N_{G}} & 0 & 0 & 0 & 0\\ \frac{\delta P_{S3}}{\delta N_{G}} & 0 & 0 & 0 & 0\\ \frac{2}{J_{T}}\cdot \frac{\delta Q_{P}}{\delta N_{G}} & -\frac{1}{J_{T}} & 0 & 0 & \frac{2}{J_{T}}\cdot \frac{\delta Q_{P}}{\delta N_{P}} \end{array}],\;B_{2}=[\begin{array}{c} \frac{1}{J_{G}}\cdot \frac{\delta Q_{G}}{\delta W_{F}}\\ \frac{2\cdot DMR}{J_{T}}\cdot \frac{\delta Q_{P}}{\delta W_{F}}\\ \frac{\delta T_{45}}{\delta W_{F}}\\ \frac{\delta P_{S3}}{\delta W_{F}}\\ \frac{2}{J_{T}}\cdot \frac{\delta Q_{P}}{\delta W_{F}} \end{array}],\;C_{2}=[\begin{array}{ccccc} 0 & 1 & 0 & 0 & 0 \end{array}]$$

and $B_{3}$ is a real constant matrix with an appropriate dimension.

### Output feedback control

The output feedback controller is utilized in *K*_1_ considering the system reference input *N*_*Pr*_ = 0. The controller uses a discrete-time form in practical applications.

$$\begin{array}{c} u_{1}\left(k\right)=K_{1}y_{1}\left(k\right) \end{array}$$ (3)

where, *y*_1_(*k*) is the output vector of the rotor system in discrete-time form, and *K*_1_ is the output feedback gain. The *K*_2_ also uses the output feedback form as follows:

$$\begin{array}{c} u_{2}\left(k\right)=u_{1}\left(k\right)+K_{2}y_{2}\left(k\right) \end{array}$$ (4)

where, *y*_2_(*k*) is the output vector of the gas generator in discrete-time form, and *K*_2_ is the output feedback gain.

The gas generator receives the piecewise control input provided as follows by considering the network-induced delay *τ*_*k*_:

u(t)=u2(k-1)kh≤t<kh+τku2(k)kh+τk≤t<(k+1)h (5)

The rotor system and the gas generator with the sampling period, [*kh*, (*k*+1)*h*], are discretized as follows by using Eq (5):

$$\begin{array}{c} \begin{array}{cc} & x_{1}\left(k+1\right)=A_{1}x_{1}\left(k\right)+B_{1}y_{2}\left(k\right)\\ & y_{1}\left(k\right)=C_{1}x_{1}\left(k\right) \end{array} \end{array}$$ (6)

where, $A_{1}=e^{A_{1}h}$, $B_{1}=\int_{0}^{h}e^{A_{1}s}dsB_{1}.$

$$\begin{array}{c} \begin{array}{cc} & x_{2}\left(k+1\right)=A_{2}x_{2}\left(k\right)+B_{21}^{k}u_{2}\left(k\right)+B_{22}^{k}u_{2}\left(k-1\right)+B_{3}w\left(k\right)\\ & y_{2}\left(k\right)=C_{2}x_{2}\left(k\right) \end{array} \end{array}$$ (7)

where, $A_{2}=e^{A_{2}h}$, $B_{21}^{k}=\int_{kh+\tau_{k}}^{kh+h}e^{A_{2}\left(kh+h-s\right)}dsB_{2}$, $B_{22}^{k}=\int_{kh}^{kh+\tau_{k}}e^{A_{2}\left(kh+h-s\right)}dsB_{2}$, $B_{3}=\int_{0}^{h}e^{A_{2}s}dsB_{3}$.

Let *m* = *kh* + *h* − *s*, then $B_{21}^{k}=\int_{0}^{h-\tau_{k}}e^{A_{2}m}dmB_{2}$, $B_{22}^{k}=\int_{h-\tau_{k}}^{h}e^{A_{2}m}dmB_{2}$. Correspondingly, *τ*_*k*_ ∈ [0, *h*]. Therefore, let $\tau_{k}=\left(1+d_{k}\right)\frac{h}{2}$, *d*_*k*_ ∈ [−1, 1]:

$$B_{21}^{k}=\int_{0}^{\frac{h}{2}-d_{k}\frac{h}{2}}e^{A_{2}m}dm{ℬ}_{2}=\underset{B_{21}}{\underset{︸}{\int_{0}^{\frac{h}{2}}e^{A_{2}m}dm{ℬ}_{2}}}+e^{A_{2}\frac{h}{2}}\underset{\bar{F}\left(d_{k}\right)}{\underset{︸}{\int_{0}^{-d_{k}\frac{h}{2}}e^{A_{2}m}dm{ℬ}_{2}}}$$

Let $F_{m}=\max_{d_{k}\in \left[-1,1\right]}{∥\bar{F}\left(d_{k}\right)∥}_{2}={∥\int_{0}^{\frac{h}{2}}e^{A_{2}m}dm∥}_{2}$, $D=F_{m}e^{A_{2}\frac{h}{2}}$, $F=\bar{F}\left(d_{k}\right)/F_{m}$, then

$$B_{21}^{k}=B_{21}+DFB_{2}.$$

Let $B_{22}=\int_{\frac{h}{2}}^{h}e^{A_{2}m}dmB_{2}$, then

$$B_{22}^{k}=B_{22}-DFB_{2}.$$

$$\begin{array}{c} \begin{array}{cc} & x_{2}\left(k+1\right)=A_{2}x_{2}\left(k\right)+\left(B_{21}+DFB_{2}\right)u_{2}\left(k\right)+\left(B_{22}-DFB_{2}\right)u_{2}\left(k-1\right)+B_{3}w\left(k\right)\\ & y_{2}\left(k\right)=C_{2}x_{2}\left(k\right) \end{array} \end{array}$$ (8)

In general, the network-induced delay in an NCCS is usually uncertain because of the network transmission and network load uncertainty. A robust *H*_∞_ control is an efficient tool to analyze these problems and deal with the uncertainty. This study aims to design the output controllers to regulate the power turbine speed in the presence of disturbances. Hence, the closed-loop output is determined by *y*_1_(*k*), and the input is an exogenous disturbance *w*(*k*). Observing Eqs (6) and (7), *x*_1_(*k*), *x*_2_(*k*), and *u*_2_(*k* − 1) are chosen as the closed-loop state vectors. Therefore, the closed-loop state-space form is provided as follows:

$$\begin{array}{c} \begin{array}{cc} & \left[\begin{array}{c} x_{1}\left(k+1\right)\\ x_{2}\left(k+1\right)\\ u_{2}\left(k\right) \end{array}\right]=\left[\begin{array}{ccc} A_{1} & B_{1}C_{2} & 0\\ O_{1} & O_{2} & O_{3}\\ K_{1}C_{1} & K_{2}C_{2} & 0 \end{array}\right]\left[\begin{array}{c} x_{1}\left(k\right)\\ x_{2}\left(k\right)\\ u_{2}\left(k-1\right) \end{array}\right]+\left[\begin{array}{c} 0\\ B_{3}\\ 0 \end{array}\right]w\\ & y_{1}\left(k\right)=C_{1}x_{1}\left(k\right) \end{array} \end{array}$$ (9)

where, $O_{1}=\left(B_{21}+DFB_{2}\right)K_{1}C_{1}$, $O_{2}=A_{2}+\left(B_{21}+DFB_{2}\right)K_{2}C_{2}$, $O_{3}=B_{22}-DFB_{2}$.

**Definition 1** *Given a certain constant γ* > 0, *the γ-suboptimal output feedback H_∞_ stabilization control laws exist for the closed-loop DEC* Eq (9), *and the disturbance attenuation degree of the system is γ*, *if there exist output feedback control laws* Eqs (3) *and* (4), *which make the DEC system* Eq (9) *as robust asymptotically stable*, *and the closed-loop output y_1_(k) and the disturbance w(k) are subject to the H_∞_ norm-bounded constraint ‖y_1_(k)‖_2_* ≤ *γ‖w(k)‖_2_ under zero initial conditions*.

The following lemmas are required to derive the sufficient conditions for robust asymptotic stability:

**Lemma 1 (Schur Complement)** *Given constant matrices* Ω_1_, Ω_2_, *and* Ω_3_, *where* $\Omega_{1}=\Omega_{1}^{T}$ *and* $\Omega_{2}=\Omega_{2}^{T}>0$, *then* $\Omega_{1}+\Omega_{3}^{T}\Omega_{2}^{-1}\Omega_{3}<0$ *if and only if*:

$$\begin{array}{c} \left[\begin{array}{cc} \Omega_{1} & \Omega_{3}^{T}\\ \Omega_{3} & -\Omega_{2} \end{array}\right]<0\;\text{or}\;\left[\begin{array}{cc} -\Omega_{2} & \Omega_{3}\\ \Omega_{3}^{T} & \Omega_{1} \end{array}\right]<0 \end{array}$$

**Lemma 2** *Given constant matrices* Ω_1_, Ω_2_, *and* Ω_3_, *where* $\Omega_{1}=\Omega_{1}^{T}$, *for any* Δ_*k*_ *subject to* $\Delta_{k}^{T}\Delta_{k}\leq I$, $\Omega_{1}+\Omega_{3}\Delta_{k}\Omega_{2}+\Omega_{2}^{T}\Delta_{k}^{T}\Omega_{3}^{T}<0$ *holds if a constant scalar α* > 0 *exists satisfying*:

$$\begin{array}{c} \Omega_{1}+\alpha^{-1}\Omega_{2}^{T}\Omega_{2}+\alpha \Omega_{3}\Omega_{3}^{T}<0 \end{array}$$

**Theorem 1** *For the closed-loop NCCS shown in* Eq (9) *with disturbances*, *given a constant γ* > 0, *the optimization problem* Eq (10) *has the feasible solution min ρ if symmetric positive-definite matrices*, *X*, *Y*, *Z*, *W*_1_, *and W*_2_, *exist with corresponding dimensions and a constant λ* > 0,

$$\begin{array}{c} \left[\begin{array}{ccccccccc} -X & 0 & 0 & 0 & {\left(B_{21}W_{1}\right)}^{T} & {\left(A_{1}X\right)}^{T} & {\left(W_{1}\right)}^{T} & {\left(B_{2}W_{1}\right)}^{T} & {\left(C_{1}X\right)}^{T}\\ 0 & -Y & 0 & 0 & {\left(A_{2}+B_{21}W_{2}\right)}^{T} & {\left(B_{1}C_{2}Y\right)}^{T} & {\left(W_{2}\right)}^{T} & {\left(B_{2}W_{2}\right)}^{T} & 0\\ 0 & 0 & -Z & 0 & {\left(B_{22}Z\right)}^{T} & 0 & 0 & -{\left(B_{2}Z\right)}^{T} & 0\\ 0 & 0 & 0 & -\rho I & B_{3}^{T} & 0 & 0 & 0 & 0\\ B_{21}W_{1} & A_{2}+B_{21}W_{2} & B_{22}Z & B_{3} & \lambda DD^{T}-Y & 0 & 0 & 0 & 0\\ A_{1}X & B_{1}C_{2}Y & 0 & 0 & 0 & -X & 0 & 0 & 0\\ W_{1} & W_{2} & 0 & 0 & 0 & 0 & -Z & 0 & 0\\ B_{2}W_{1} & B_{2}W_{2} & -B_{2}Z & 0 & 0 & 0 & 0 & -\lambda I & 0\\ C_{1}X & 0 & 0 & 0 & 0 & 0 & 0 & 0 & -I \end{array}\right]<0 \end{array}$$ (10)

*The γ-optimal output feedback H*_∞_ *control laws then exist*, *and the gain matrices are provided as follows*:

$$\begin{array}{c} K_{1}=\frac{W_{1}}{C_{1}X},K_{2}=\frac{W_{2}}{C_{2}Y} \end{array}$$ (11)

The optimal solution ρ* *becomes available*, *and* $\min\gamma =\sqrt{\rho^{*}}$.

***Proof***: A quadratic Lyapunov function in discrete-time form is defined as follows to derive the sufficient conditions for robust asymptotic stability of Eq (9):

$$\begin{array}{c} V_{k}=x_{1}^{T}\left(k\right)Px_{1}\left(k\right)+x_{2}^{T}\left(k\right)Qx_{2}\left(k\right)+u_{2}^{T}\left(k-1\right)Su_{2}\left(k-1\right) \end{array}$$ (12)

where, *P*, *Q*, and *S* are the symmetric positive-definite matrices with corresponding dimensions.

The Δ*V*_*k*_ can be obtained by the following equation by taking the Lyapunov function derivative:

$$\begin{array}{c} \begin{array}{cc} \Delta V_{k} & =V_{k+1}-V_{k}\\ & =x_{1}^{T}\left(k+1\right)Px_{1}\left(k+1\right)+x_{2}^{T}\left(k+1\right)Qx_{2}\left(k+1\right)+u_{2}^{T}\left(k\right)Su_{2}\left(k\right)\\ & -x_{1}^{T}\left(k\right)Px_{1}\left(k\right)-x_{2}^{T}\left(k\right)Qx_{2}\left(k\right)-u_{2}^{T}\left(k-1\right)Su_{2}\left(k-1\right)\\ & =\left[\begin{array}{cccc} x_{1}^{T}\left(k\right) & x_{2}^{T}\left(k\right) & u_{2}^{T}\left(k-1\right) & w^{T}\left(k\right) \end{array}\right]\cdot \Xi \cdot \left[\begin{array}{c} x_{1}\left(k\right)\\ x_{2}\left(k\right)\\ u_{2}\left(k-1\right)\\ w(k) \end{array}\right] \end{array} \end{array}$$ (13)

where,

$$\begin{array}{c} \begin{array}{cc} \Xi & =\left[\begin{array}{cccc} A_{1}^{T}PA_{1}+{\left(K_{1}C_{1}\right)}^{T}SK_{1}C_{1}-P+O_{1}^{T}QO_{1} & A_{1}^{T}PB_{1}C_{2}+{\left(K_{1}C_{1}\right)}^{T}SK_{2}C_{2}+O_{1}^{T}QO_{2} & O_{1}^{T}QO_{3} & O_{1}^{T}QB_{3}\\ {\left(B_{1}C_{2}\right)}^{T}PA_{1}+{\left(K_{2}C_{2}\right)}^{T}SK_{1}C_{1}+O_{2}^{T}QO_{1} & {\left(B_{1}C_{2}\right)}^{T}PB_{1}C_{2}+{\left(K_{2}C_{2}\right)}^{T}SK_{2}C_{2}-Q+O_{2}^{T}O_{2} & O_{2}^{T}QO_{3} & O_{2}^{T}QB_{3}\\ O_{3}^{T}QO_{1} & O_{3}^{T}QO_{2} & -S+O_{3}^{T}QO_{3} & O_{3}^{T}QB_{3}\\ B_{3}^{T}QO_{1} & B_{3}^{T}QO_{2} & B_{3}^{T}QO_{3} & B_{3}^{T}QB_{3} \end{array}\right]\\ & =\left[\begin{array}{cccc} A_{1}^{T}PA_{1}+{\left(K_{1}C_{1}\right)}^{T}SK_{1}C_{1}-P & A_{1}^{T}PB_{1}C_{2}+{\left(K_{1}C_{1}\right)}^{T}SK_{2}C_{2} & 0 & 0\\ {\left(B_{1}C_{2}\right)}^{T}PA_{1}+{\left(K_{2}C_{2}\right)}^{T}SK_{1}C_{1} & {\left(B_{1}C_{2}\right)}^{T}PB_{1}C_{2}+{\left(K_{2}C_{2}\right)}^{T}SK_{2}C_{2}-Q & 0 & 0\\ 0 & 0 & -S & 0\\ 0 & 0 & 0 & 0 \end{array}\right]+\left[\begin{array}{c} O_{1}^{T}\\ O_{2}^{T}\\ O_{3}^{T}\\ B_{3}^{T} \end{array}\right]\cdot Q\cdot \left[\begin{array}{cccc} O_{1} & O_{2} & O_{3} & B_{3} \end{array}\right] \end{array} \end{array}$$

The performance index function can be defined as follows considering the closed-loop robust stability with disturbance:

$$\begin{array}{c} J=\sum_{k=0}^{\infty }\left(y_{1}^{T}\left(k\right)y_{1}\left(k\right)-\gamma^{2}w^{T}\left(k\right)w\left(k\right)\right) \end{array}$$ (14)

The following condition must be satisfied if the disturbance attenuation degree is *γ* > 0:

$$\begin{array}{c} ∥y_{1}{\left(k\right)∥}_{2}\leq \gamma {∥w\left(k\right)∥}_{2} \end{array}$$ (15)

(i.e., *J* ≤ 0). The following equation is obtained for the system with a non-zero disturbance satisfying *w*(*t*) ∈ [0, ∞) and zero initial conditions:

$$\begin{array}{c} \begin{array}{l} J\leq \overset{\infty }{\underset{k=0}{\sum }}\left(y_{1}^{T}\left(k\right)y_{1}\left(k\right)-\gamma^{2}w^{T}\left(k\right)w\left(k\right)+\Delta V_{k}\right)\\ \;=\left[\begin{array}{cccc} x_{1}{\left(k\right)}^{T} & x_{2}{\left(k\right)}^{T} & u_{2}{\left(k-1\right)}^{T} & w^{T}\left(k\right) \end{array}\right]\cdot \underset{\Gamma }{\underset{︸}{\left(\left[\begin{array}{cccc} C_{1}^{T}C_{1} & 0 & 0 & 0\\ 0 & 0 & 0 & 0\\ 0 & 0 & 0 & 0\\ 0 & 0 & 0 & -\gamma^{2}I \end{array}\right]+\Xi \right)}}\cdot \left[\begin{array}{c} x_{1}\left(k\right)\\ x_{2}\left(k\right)\\ u_{2}\left(k-1\right)\\ w\left(k\right) \end{array}\right] \end{array} \end{array}$$ (16)

where,

$$\begin{array}{c} \begin{array}{cc} \Gamma & =\left[\begin{array}{cccc} A_{1}^{T}PA_{1}+{\left(K_{1}C_{1}\right)}^{T}SK_{1}C_{1}-P+C_{1}^{T}C_{1} & A_{1}^{T}PB_{1}C_{2}+{\left(K_{1}C_{1}\right)}^{T}SK_{2}C_{2} & 0 & 0\\ {\left(B_{1}C_{2}\right)}^{T}PA_{1}+{\left(K_{2}C_{2}\right)}^{T}SK_{1}C_{1} & {\left(B_{1}C_{2}\right)}^{T}PB_{1}C_{2}+{\left(K_{2}C_{2}\right)}^{T}SK_{2}C_{2}-Q & 0 & 0\\ 0 & 0 & -S & 0\\ 0 & 0 & 0 & -\gamma^{2}I \end{array}\right]+\left[\begin{array}{c} O_{1}^{T}\\ O_{2}^{T}\\ O_{3}^{T}\\ B_{3}^{T} \end{array}\right]\cdot Q\cdot \left[\begin{array}{cccc} O_{1} & O_{2} & O_{3} & B_{3} \end{array}\right]\\ & =\left[\begin{array}{ccccc} A_{1}^{T}PA_{1}+{\left(K_{1}C_{1}\right)}^{T}SK_{1}C_{1}-P+C_{1}^{T}C_{1} & A_{1}^{T}PB_{1}C_{2}+{\left(K_{1}C_{1}\right)}^{T}SK_{2}C_{2} & 0 & 0 & O_{1}^{T}\\ {\left(B_{1}C_{2}\right)}^{T}PA_{1}+{\left(K_{2}C_{2}\right)}^{T}SK_{1}C_{1} & {\left(B_{1}C_{2}\right)}^{T}PB_{1}C_{2}+{\left(K_{2}C_{2}\right)}^{T}SK_{2}C_{2}-Q & 0 & 0 & O_{2}^{T}\\ 0 & 0 & -S & 0 & O_{3}^{T}\\ 0 & 0 & 0 & -\gamma^{2}I & B_{3}^{T}\\ O_{1} & O_{2} & O_{3} & B_{3} & -Q^{-1} \end{array}\right]\\ & =\left[\begin{array}{ccccccc} C_{1}^{T}C_{1}-P & 0 & A_{1}^{T} & {\left(K_{1}C_{1}\right)}^{T} & 0 & 0 & O_{1}^{T}\\ 0 & -Q & {\left(B_{1}C_{2}\right)}^{T} & {\left(K_{2}C_{2}\right)}^{T} & 0 & 0 & O_{2}^{T}\\ A_{1} & B_{1}C_{2} & -P^{-1} & 0 & 0 & 0 & 0\\ K_{1}C_{1} & K_{2}C_{2} & 0 & -S^{-1} & 0 & 0 & 0\\ 0 & 0 & 0 & 0 & -S & 0 & O_{3}^{T}\\ 0 & 0 & 0 & 0 & 0 & -\gamma^{2}I & B_{3}^{T}\\ O_{1} & O_{2} & 0 & 0 & O_{3} & B_{3} & -Q^{-1} \end{array}\right]\\ & =\left[\begin{array}{ccccccc} C_{1}^{T}C_{1}-P & 0 & 0 & 0 & O_{1}^{T} & A_{1}^{T} & {\left(K_{1}C_{1}\right)}^{T}\\ 0 & -Q & 0 & 0 & Q_{2}^{T} & {\left(B_{1}C_{2}\right)}^{T} & {\left(K_{2}C_{2}\right)}^{T}\\ 0 & 0 & -S & 0 & Q_{3}^{T} & 0 & 0\\ 0 & 0 & 0 & -\gamma^{2}I & B_{3}^{T} & 0 & 0\\ O_{1} & O_{2} & O_{3} & B_{3} & -Q^{-1} & 0 & 0\\ A_{1} & B_{1}C_{2} & 0 & 0 & 0 & -P^{-1} & 0\\ K_{1}C_{1} & K_{2}C_{2} & 0 & 0 & 0 & 0 & -S^{-1} \end{array}\right] \end{array} \end{array}$$

Substituting *O*_1_, *O*_2_, and *O*_3_ into *Γ*,

$$\begin{array}{c} \begin{array}{cc} \Gamma & =\left[\begin{array}{ccccccc} C_{1}^{T}C_{1}-P & 0 & 0 & 0 & {\left(\left(B_{21}+DFB_{2}\right)K_{1}C_{1}\right)}^{T} & A_{1}^{T} & {\left(K_{1}C_{1}\right)}^{T}\\ 0 & -Q & 0 & 0 & {\left(A_{2}+\left(B_{21}+DFB_{2}\right)K_{2}C_{2}\right)}^{T} & {\left(B_{1}C_{2}\right)}^{T} & {\left(K_{2}C_{2}\right)}^{T}\\ 0 & 0 & -S & 0 & {\left(B_{21}-DFB_{2}\right)}^{T} & 0 & 0\\ 0 & 0 & 0 & -\gamma^{2}I & B_{3}^{T} & 0 & 0\\ \left(B_{21}+DFB_{2}\right)K_{1}C_{1} & A_{2}+\left(B_{21}+DFB_{2}\right)K_{2}C_{2} & B_{21}-DFB_{2} & B_{3} & -Q^{-1} & 0 & 0\\ A_{1} & B_{1}C_{2} & 0 & 0 & 0 & -P^{-1} & 0\\ K_{1}C_{1} & K_{2}C_{2} & 0 & 0 & 0 & 0 & -S^{-1} \end{array}\right]\\ & =\left[\begin{array}{ccccccc} C_{1}^{T}C_{1}-P & 0 & 0 & 0 & {\left(B_{21}K_{1}C_{1}\right)}^{T} & A_{1}^{T} & {\left(K_{1}C_{1}\right)}^{T}\\ 0 & -Q & 0 & 0 & (A_{2}+{\left(B_{21}K_{2}C_{2}\right)}^{T} & {\left(B_{1}C_{2}\right)}^{T} & {\left(K_{2}C_{2}\right)}^{T}\\ 0 & 0 & -S & 0 & B_{22}^{T} & 0 & 0\\ 0 & 0 & 0 & -\gamma^{2}I & B_{3}^{T} & 0 & 0\\ B_{21}K_{1}C_{1} & A_{2}+B_{21}K_{2}C_{2} & B_{22} & B_{3} & -Q^{-1} & 0 & 0\\ A_{1} & B_{1}C_{2} & 0 & 0 & 0 & -P^{-1} & 0\\ K_{1}C_{1} & K_{2}C_{2} & 0 & 0 & 0 & 0 & -S^{-1} \end{array}\right]\\ & \;+\left[\begin{array}{c} 0\\ 0\\ 0\\ 0\\ D\\ 0\\ 0 \end{array}\right]F\left[\begin{array}{ccccccc} B_{2}K_{1}C_{1} & B_{2}K_{2}C_{2} & -B_{2} & 0 & 0 & 0 & 0 \end{array}\right]+\left[\begin{array}{c} {\left(B_{2}K_{1}C_{1}\right)}^{T}\\ {\left(B_{2}K_{2}C_{2}\right)}^{T}\\ -B_{2}^{T}\\ 0\\ 0\\ 0\\ 0 \end{array}\right]F^{T}\left[\begin{array}{ccccccc} 0 & 0 & 0 & 0 & D^{T} & 0 & 0 \end{array}\right] \end{array} \end{array}$$

Using Lemma 2, a constant *λ* > 0 exists to satisfy:

$$\begin{array}{c} \begin{array}{cc} \Gamma & =\left[\begin{array}{ccccccc} C_{1}^{T}C_{1}-P & 0 & 0 & 0 & {\left(B_{21}K_{1}C_{1}\right)}^{T} & A_{1}^{T} & {\left(K_{1}C_{1}\right)}^{T}\\ 0 & -Q & 0 & 0 & (A_{2}+{\left(B_{21}K_{2}C_{2}\right)}^{T} & {\left(B_{1}C_{2}\right)}^{T} & {\left(K_{2}C_{2}\right)}^{T}\\ 0 & 0 & -S & 0 & B_{22}^{T} & 0 & 0\\ 0 & 0 & 0 & -\gamma^{2}I & B_{3}^{T} & 0 & 0\\ B_{21}K_{1}C_{1} & A_{2}+B_{21}K_{2}C_{2} & B_{22} & B_{3} & -Q^{-1} & 0 & 0\\ A_{1} & B_{1}C_{2} & 0 & 0 & 0 & -P^{-1} & 0\\ K_{1}C_{1} & K_{2}C_{2} & 0 & 0 & 0 & 0 & -S^{-1} \end{array}\right]+\lambda \left[\begin{array}{c} 0\\ 0\\ 0\\ 0\\ D\\ 0\\ 0 \end{array}\right]\left[\begin{array}{ccccccc} 0 & 0 & 0 & 0 & D^{T} & 0 & 0 \end{array}\right]\\ & \;+\lambda^{-1}\left[\begin{array}{c} {\left(B_{2}K_{1}C_{1}\right)}^{T}\\ {\left(B_{2}K_{2}C_{2}\right)}^{T}\\ -B_{2}^{T}\\ 0\\ 0\\ 0\\ 0 \end{array}\right]\left[\begin{array}{ccccccc} B_{2}K_{1}C_{1} & B_{2}K_{2}C_{2} & -B_{2} & 0 & 0 & 0 & 0 \end{array}\right]<0 \end{array} \end{array}$$

Therefore, *J* ≤ 0. Using Lemma 1,

$$\begin{array}{c} \begin{array}{cc} \Gamma & =\left[\begin{array}{cccccccc} C_{1}^{T}C_{1}-P & 0 & 0 & 0 & {\left(B_{21}K_{1}C_{1}\right)}^{T} & A_{1}^{T} & {\left(K_{1}C_{1}\right)}^{T} & {\left(B_{2}K_{1}C_{1}\right)}^{T}\\ 0 & -Q & 0 & 0 & (A_{2}+{\left(B_{21}K_{2}C_{2}\right)}^{T} & {\left(B_{1}C_{2}\right)}^{T} & {\left(K_{2}C_{2}\right)}^{T} & {\left(B_{2}K_{2}C_{2}\right)}^{T}\\ 0 & 0 & -S & 0 & B_{22}^{T} & 0 & 0 & -B_{2}^{T}\\ 0 & 0 & 0 & -\gamma^{2}I & B_{3}^{T} & 0 & 0 & 0\\ B_{21}K_{1}C_{1} & A_{2}+B_{21}K_{2}C_{2} & B_{22} & B_{3} & \lambda DD^{T}-Q^{-1} & 0 & 0 & 0\\ A_{1} & B_{1}C_{2} & 0 & 0 & 0 & -P^{-1} & 0 & 0\\ K_{1}C_{1} & K_{2}C_{2} & 0 & 0 & 0 & 0 & -S^{-1} & 0\\ B_{2}K_{1}C_{1} & B_{2}K_{2}C_{2} & -B_{2} & 0 & 0 & 0 & 0 & -\lambda I \end{array}\right]\\ & =\left[\begin{array}{ccccccccc} -P & 0 & 0 & 0 & {\left(B_{21}K_{1}C_{1}\right)}^{T} & A_{1}^{T} & {\left(K_{1}C_{1}\right)}^{T} & {\left(B_{2}K_{1}C_{1}\right)}^{T} & C_{1}^{T}\\ 0 & -Q & 0 & 0 & {\left(A_{2}+B_{21}K_{2}C_{2}\right)}^{T} & {\left(B_{1}C_{2}\right)}^{T} & {\left(K_{2}C_{2}\right)}^{T} & {\left(B_{2}K_{2}C_{2}\right)}^{T} & 0\\ 0 & 0 & -S & 0 & B_{22}^{T} & 0 & 0 & -B_{2}^{T} & 0\\ 0 & 0 & 0 & -\gamma^{2}I & B_{3}^{T} & 0 & 0 & 0 & 0\\ B_{21}K_{1}C_{1} & A_{2}+B_{21}K_{2}C_{2} & B_{22} & B_{3} & \lambda DD^{T}-Q^{-1} & 0 & 0 & 0 & 0\\ A_{1} & B_{1}C_{2} & 0 & 0 & 0 & -P^{-1} & 0 & 0 & 0\\ K_{1}C_{1} & K_{2}C_{2} & 0 & 0 & 0 & 0 & -S^{-1} & 0 & 0\\ B_{2}K_{1}C_{1} & B_{2}K_{2}C_{2} & -B_{2} & 0 & 0 & 0 & 0 & -\lambda I & 0\\ C_{1} & 0 & 0 & 0 & 0 & 0 & 0 & 0 & -I \end{array}\right]<0 \end{array} \end{array}$$ (17)

Eq (10) can then be obtained by pre- and post-multiplying Eq (17) by *diag*(*P*^−1^, *Q*^−1^, *S*^−1^, *I*, *I*, *I*, *I*, *I*, *I*) and letting *X* = *P*^−1^, *Y* = *Q*^−1^, *Z* = *S*^−1^, *W*_1_ = *K*_1_*C*_1_, and *W*_2_ = *K*_2_*C*_2_.

**Algorithm 1** *We will now provide the algorithm for the controller design*.

*Step 1*: *The continuous closed-loop system parameters are derived based on* Fig 3.

*Step 2*: *The continuous system parameters are discretized*.

*Step 3*: *The convex optimization problem* (Eq (10)) *is solved to obtain the feasible solutions in terms of positive definite matrices X*, *Y*, *and Z and matrices W*_1_, *W*_2_, and ρ*.

*Step 4*: *The controller parameters K*_1_, *K*_2_, *and γ are derived based on Theorem* 1.

## Simulation Examples

This section presents the effectiveness evaluation of the proposed method under two kinds of simulation in the GE T700 turboshaft gas turbine engine DEC control systems. The rotor system in continuous time form is provided as follows:

$$\begin{array}{c} \begin{array}{cc} & \left[\begin{array}{c} {\dot{N}}_{P}\\ {\dot{N}}_{MR}\\ {\dot{Q}}_{MR} \end{array}\right]=\left[\begin{array}{ccc} 0 & 0 & -285.7143\\ 0 & -0.4533 & 9.0662\\ 5.2650 & -5.2131 & -42.5958 \end{array}\right]\left[\begin{array}{c} N_{P}\\ N_{MR}\\ Q_{MR} \end{array}\right]+\left[\begin{array}{c} 571.4286\\ 0\\ 82.5714 \end{array}\right]Q_{S}\\ & N_{P}=\left[\begin{array}{ccc} 1 & 0 & 0 \end{array}\right]\left[\begin{array}{c} N_{P}\\ N_{MR}\\ Q_{MR} \end{array}\right] \end{array} \end{array}$$

The gas generator model is given as:

$$\begin{array}{c} \begin{array}{cc} & \left[\begin{array}{c} {\dot{N}}_{G}\\ {\dot{Q}}_{S}\\ {\dot{T}}_{45}\\ {\dot{P}}_{S3}\\ {\dot{N}}_{P} \end{array}\right]=\left[\begin{array}{ccccc} -126.8 & 27.04 & 12.36 & 22.17 & 16.72\\ 54.67 & 57.21 & -77.02 & -76.21 & 50.81\\ -336.6 & 223.3 & -130.7 & -83.32 & 172.1\\ 161.2 & 2.459 & -21.8 & -63.09 & 1.799\\ 62.42 & -73.55 & -104.2 & -91.44 & -102.3 \end{array}\right]\left[\begin{array}{c} N_{G}\\ Q_{S}\\ T_{45}\\ P_{S3}\\ N_{P} \end{array}\right]+\left[\begin{array}{c} -11.7\\ 44.24\\ 53.56\\ 17.45\\ 59.35 \end{array}\right]W_{F}+\left[\begin{array}{c} 0.02\\ 0.02\\ 0.02\\ 0.02\\ 0.02 \end{array}\right]w\\ & Q_{S}=\left[\begin{array}{ccccc} 0 & 1 & 0 & 0 & 0 \end{array}\right]\left[\begin{array}{c} N_{G}\\ Q_{S}\\ T_{45}\\ P_{S3}\\ N_{P} \end{array}\right] \end{array} \end{array}$$

The coefficients after the discretization are provided as follows:

$$\begin{array}{c} \begin{array}{c} A_{1}=\left[\begin{array}{ccc} 0.9352 & 0.0640 & -2.2670\\ 0.0021 & 0.9934 & 0.0718\\ 0.0418 & -0.0413 & 0.5952 \end{array}\right],B_{1}=\left[\begin{array}{c} 4.5703\\ 0.0362\\ 0.7847 \end{array}\right],C_{1}=\left[\begin{array}{ccc} 1 & 0 & 0 \end{array}\right],\\ A_{2}=\left[\begin{array}{ccccc} 0.3927 & 0.1572 & -0.0524 & 0.0010 & 0.0772\\ 0.6985 & 1.0228 & -0.4391 & -0.4080 & 0.0411\\ -0.5933 & 0.5866 & -0.1759 & -0.8311 & 0.3484\\ 0.8005 & 0.0546 & -0.0665 & 0.6367 & 0.0187\\ 0.1142 & -0.8350 & 0.0242 & 0.0769 & 0.0953 \end{array}\right],B_{21}=\left[\begin{array}{c} -0.0153\\ 0.1908\\ 0.3524\\ 0.0502\\ 0.1099 \end{array}\right],B_{22}=\left[\begin{array}{c} 0.0206\\ 0.0920\\ 0.2553\\ 0.0138\\ -0.1128 \end{array}\right],B_{3}=\left[\begin{array}{c} 0.1213\\ 0.0715\\ -0.1214\\ 0.1019\\ 0.0328 \end{array}\right],\\ C_{2}=\left[\begin{array}{ccccc} 0 & 1 & 0 & 0 & 0 \end{array}\right],\\ D=\left[\begin{array}{ccccc} 0.0036 & 0.0007 & 0 & 0.0002 & 0.0004\\ 0.0023 & 0.0071 & -0.0020 & -0.0019 & 0.0007\\ -0.0048 & 0.0041 & 0.0018 & -0.0034 & 0.0029\\ 0.0036 & 0.0001 & -0.0004 & 0.0049 & 0\\ 0.0014 & -0.0030 & -0.0011 & -0.0009 & 0.0029 \end{array}\right]. \end{array} \end{array}$$

The feasible solution of Eq (10) can be calculated by using the LMI toolbox in MATLAB as follows:

$$\begin{array}{c} \begin{array}{c} \rho^{*}=0.8728,\gamma =0.9342,\\ X=\left[\begin{array}{ccc} 0.4456 & -0.0114 & 0.0580\\ -0.0114 & 0.0050 & -0.0009\\ 0.0580 & -0.0009 & 0.0189 \end{array}\right],Y=\left[\begin{array}{ccccc} 0.0002 & -0.0001 & 0.0005 & -0.0002 & 0.0001\\ -0.0001 & 0.0005 & 0.0009 & 0.0001 & -0.0001\\ 0.0005 & 0.0009 & 0.0042 & -0.0014 & -0.0001\\ -0.0002 & 0.0001 & -0.0014 & 0.0025 & 0.0002\\ 0.0001 & -0.0001 & -0.0001 & 0.0002 & 0.0165 \end{array}\right],Z=10^{-12}\times 9.8434,\\ W_{1}=10^{-11}\times \left[\begin{array}{ccc} -0.2685 & 0.0043 & -0.0068 \end{array}\right],W_{2}=10^{-11}\times \left[\begin{array}{ccccc} -0.0129 & 0.0432 & 0.0429 & 0.0072 & 0.2365 \end{array}\right],\\ K_{1}=10^{-12}\times -5.9421,K_{2}=10^{-10}\times -4.0554. \end{array} \end{array}$$

### Numerical simulation

#### Simulated setup

The DEC system is simulated herein by using the *TrueTime* network simulation software written under MATLAB/Simulink [27]. The real-time information in both control loops are transmitted via the same communication network with a sampling period of *h* = 0.01*s*. Let us assume that the two network-induced delays are both equivalent to *τ*_*k*_, which is time varying and not longer than the sampling period (i.e., *τ*_*k*_ ∈ [0, *h*]). Given the initial conditions as *x*_1_(0)=[1 0.2 0.2]^*T*^, *x*_2_(0)=[0.9000 0.4189 0.7843 0.6498 1.0000]^*T*^, the simulation time is *T* = 20*s*. Meanwhile, *N*_*Pr*_ is a unit step input at *t* = 1*s*.

#### Simulation results

**Fig 4:**
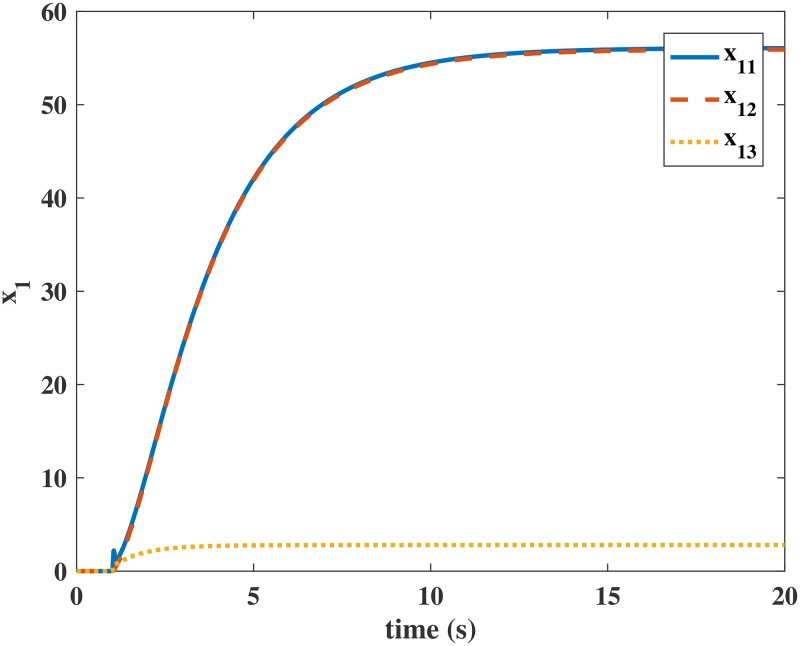
Response of *x*_1_ in the numerical simulation.

**Fig 5:**
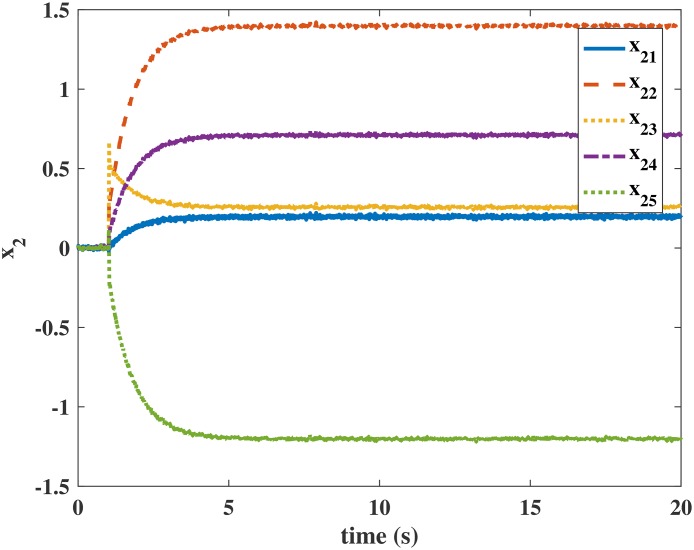
Response of *x*_2_ in the numerical simulation.

**Fig 6:**
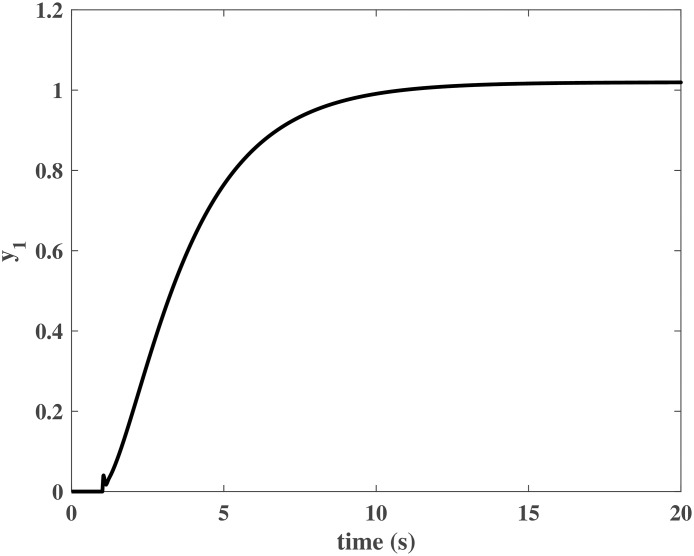
Response of *y*_1_ in the numerical simulation.

**Fig 7:**
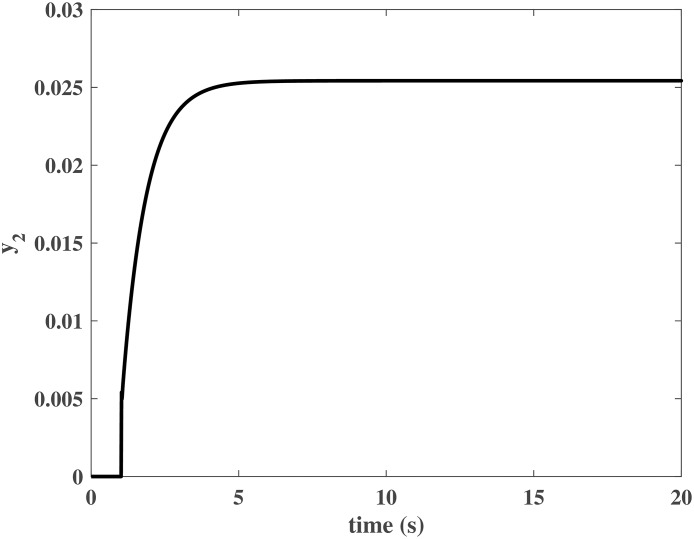
Response of *y*_2_ in the numerical simulation.

**Fig 8:**
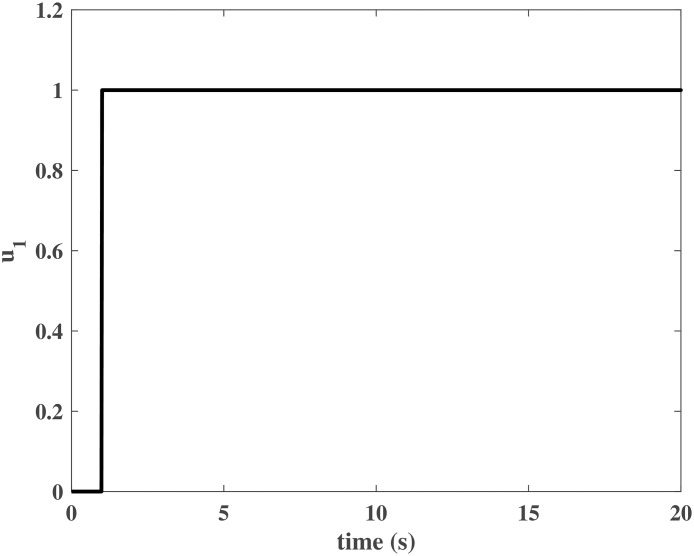
Controller output *u*_1_ in the numerical simulation.

**Fig 9:**
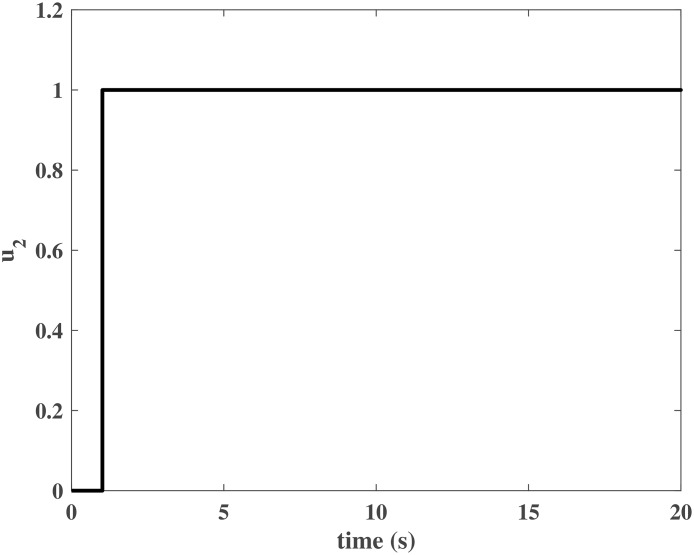
Controller output *u*_2_ in the numerical simulation.

Figs 4 and 5 present the responses of the state variables in the closed-loop system under uncertain disturbances. Figs 6 and 7 show the responses of *N*_*P*_ in the rotor system and *Q*_*S*_ in the gas generator. The closed-loop system can be asymptotically stable without any steady error under the transmission delay. Meanwhile, Figs 8 and 9 illustrate that the gas generator control loop (inner loop) is much faster than the rotor system control loop (outer loop).

### Hardware-in-loop simulation

#### Testbed description

**Fig 10:**
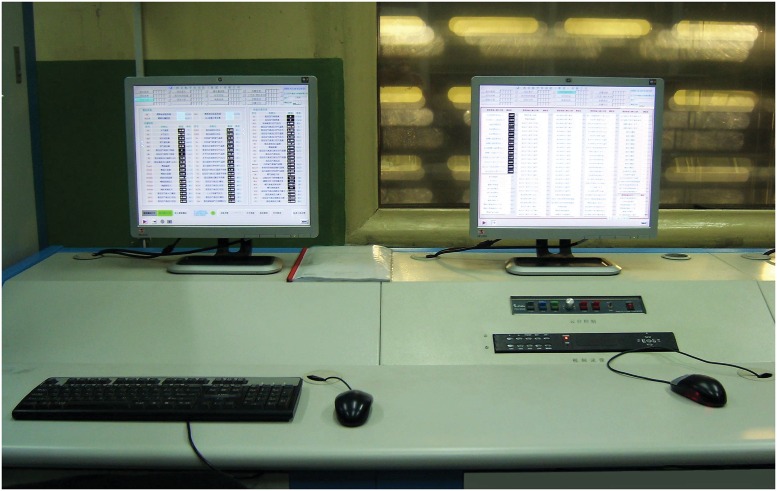
HIL system: monitors and gas generator and rotor system models.

**Fig 11:**
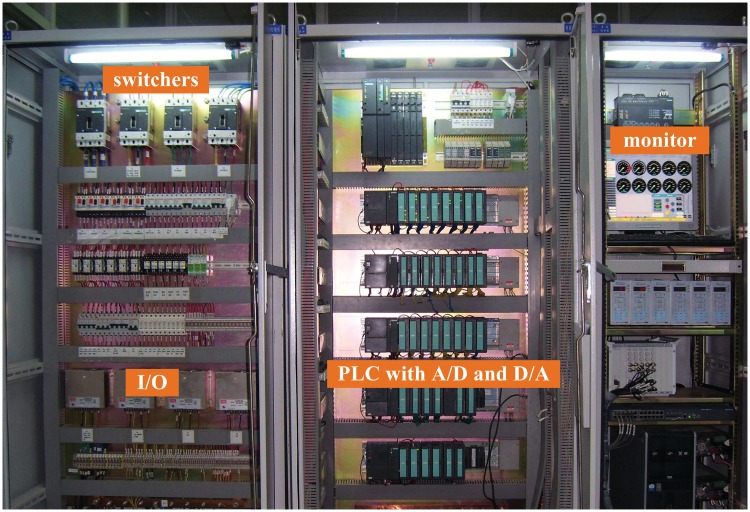
HIL system: DEC system.

**Fig 12:**
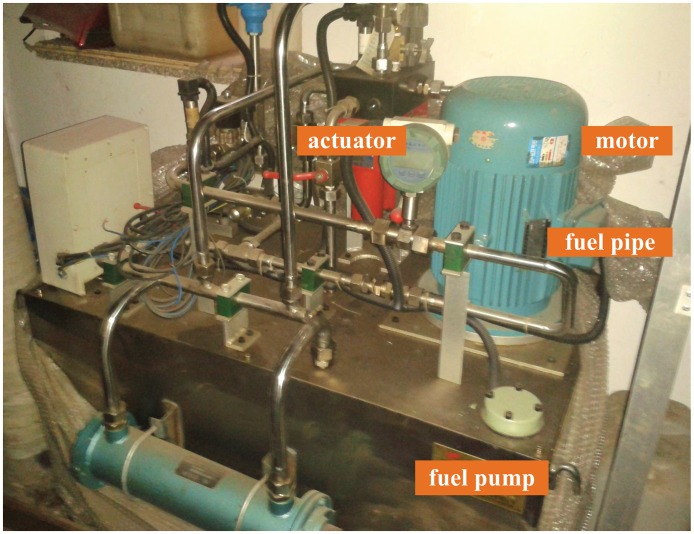
HIL system: actuator and fuel supply system.

The DEC system in this experiment was tested by using the hardware-in-loop (HIL) simulation testbed in Figs 10, 11 and 12. The left computer in Fig 10 was used as the simulation result storage installed in the GE T700 turboshaft engine model. The right computer was utilized as the manipulating interface and a monitor to watch the simulation results on time. Fig 11 shows the DEC control system configuration. The controller used was a Siemens PLC S7-300 Serial. Fig 12 shows the actuator and fuel supply system. The initial conditions, sampling period, and delay were similar to those in the numerical simulation.

#### Experimental results

**Fig 13:**
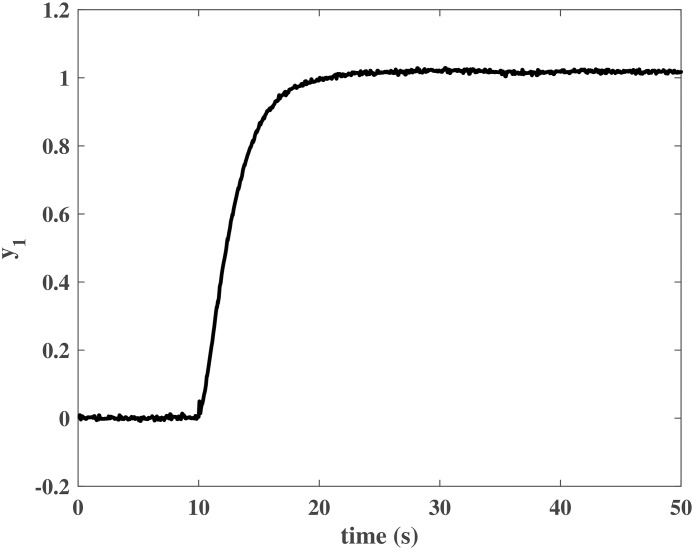
Response of *y*_1_ in the HIL simulation.

**Fig 14:**
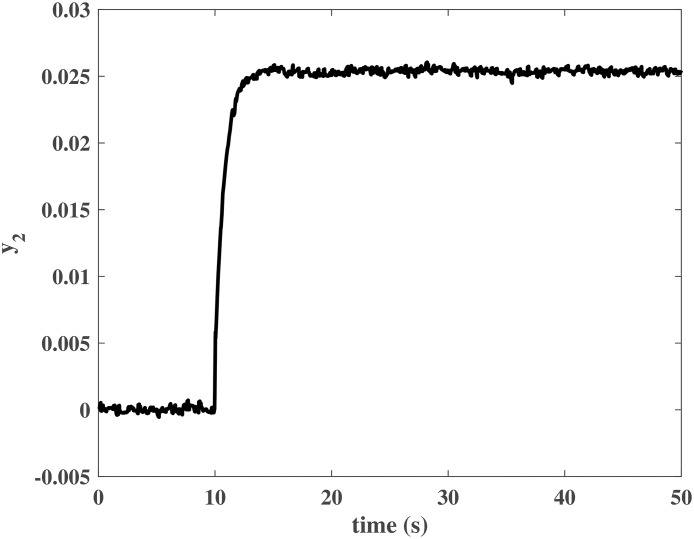
Response of *y*_2_ in the HIL simulation.

**Fig 15:**
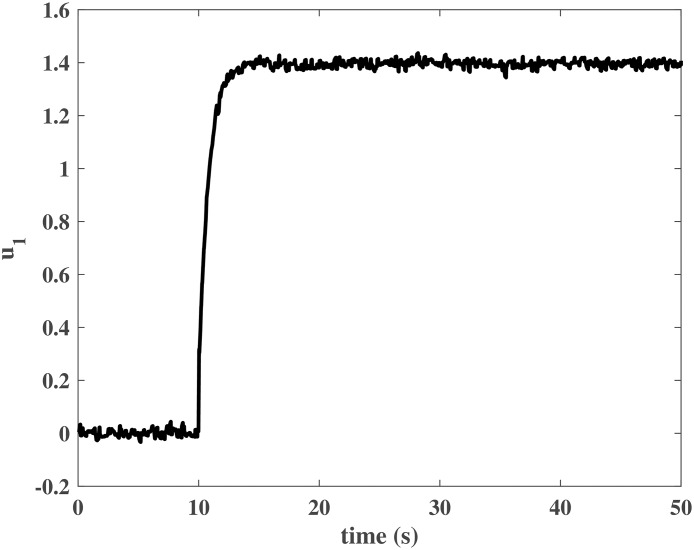
Controller output *u*_1_ in the HIL simulation.

**Fig 16:**
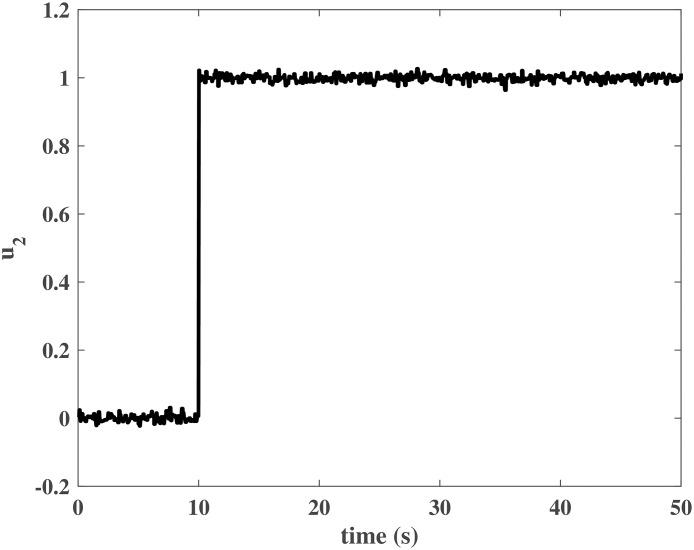
Controller output *u*_2_ in the HIL simulation.

The initial values of the closed-loop system were similar to those in the numerical simulation. The simulation time was *T* = 50*s*, and *N*_*P*_ was a unit step input at *t* = 10*s*. Figs 13 to 16 show the control effort under the disturbances. The response of the rotor system output can be fast to reach the desired value without any steady error. In other words, the closed-loop system can achieve a good robust performance when the NCCS has network-induced delays by using the proposed DEC system design method.

## Conclusions and Future Work

This study considered the novel robust *H*_∞_ distributed engine control problem to guarantee the engine performance under network-induced delays and uncertain disturbances. A partially distributed control system architecture of a typical turboshaft engine was also described accordingly. This distributed architecture can be transformed into a networked cascade control system. The output feedback controllers were designed to robustly asymptotically stabilize the closed-loop system under network-induced delays and uncertain disturbances. The sufficient conditions for asymptotic stability were derived based on the Lyapunov stability and the LMI approach. The controller design problem under consideration is solvable if the LMIs were feasible. Both numerical and hardware-in-loop simulation examples were provided to show the effectiveness of the approach. One of our future research topics would be the DEC system with simultaneous packet dropout and network-induced delays, where the latest delay-dependent techniques can be employed.
